# Influence of milking frequency on genetic parameters associated with the milk production in the first and second lactations of Iranian Holstein dairy cows using random regression test day models

**DOI:** 10.1186/s40781-016-0087-3

**Published:** 2016-02-01

**Authors:** Moslem Moghbeli Damane, Masood Asadi Fozi, Ahmad Ayatollahi Mehrgardi

**Affiliations:** Department of Animal Science, Faculty of Agriculture, University of Jiroft, Jiroft, Iran; Department of Animal Science, Faculty of Agriculture, Shahid Bahonar University of Kerman, Kerman, Iran

**Keywords:** Genetic parameters, Random regression, Milk production, Milking frequency, Iranian Holstein cows

## Abstract

**Background:**

The milk yield can be affected by the frequency of milking per day, in dairy cows. Previous studies have shown that the milk yield is increased by 6–25 % per lactation when the milking frequency is increased from 2 to 3 times per day while the somatic cell count is decreased. To investigate the effect of milking frequency (3X vs. 4X) on milk yield and it’s genetic parameters in the first and second lactations of the Iranian Holstein dairy cows, a total of 142,604 test day (TD) records of milk yield were measured on 20,762 cows.

**Results:**

Heritability estimates of milk yield were 0.25 and 0.19 for 3X milking frequency and 0.34 and 0.26 for 4X milking frequency throughout the first and second lactations, respectively. Repeatability estimates of milk yield were 0.70 and 0.71 for 3X milking frequency and 0.76 and 0.77 for 4X milking frequency, respectively. In comparison with 3X milking frequency, the milk yield of the first and second lactations was increased by 11.6 and 12.2 %, respectively when 4X was used (*p* < 0.01).

**Conclusions:**

Results of this research demonstrated that increasing milking frequency led to an increase in heritability and repeatability of milk yield. The current investigation provided clear evidences for the benefits of using 4X milking frequency instead of 3X in Iranian Holstein dairy cows.

## Background

The purpose of animal breeding is to genetically enhance the livestock production where more efficient animals are produced to guard against future circumstances. An important method for maximizing response to selection program is the accurate prediction of breeding values of animal [34]. In dairy cows, to implement an efficient breeding program, the estimates of genetic parameters for the production traits are required. In addition, to predict expected selection response and to achieve the predicted breeding value by the mixed model procedures, the accurate heritability, repeatability and correlation estimates are required. Various test day (TD) models have been recommended for genetic evaluation of dairy cows such as multiple-trait models, covariance function models and random regression models [33]. However, in many countries, the random regression model (RRM) has been widely demonstrated to increase the accuracy of breeding value predictions [31]. The use of RRM makes it possible to study changes in TD records over time and a better understanding of lactation genetics [32].

It has been found that the milk yield could be affected by the frequency of milking cows per day [16]. Erdman [14] demonstrated that the increase in milk yield due to the increased milk frequency is fixed, and is not dependent to the level of milk production at the time of increase milking frequency. In high producing dairy cows, twice a day milking interval of 10–14 h and 12–14 h are suggested, whereas, once a day or skipping milking is not acceptable, since it may arise several problems such as an increased in the somatic cell counts as well as poor udder health [30]. By increasing milking frequency from 2 to 3 times per day, an increase in milk yield from 6 to 25 % per lactation is observed [11, 14, 22] and there is also a decrease in somatic cell counts [17]. Moreover, Armstrong [1] reported that the milk production is increased by 8–12 % when four times (4X) a day milking is applied instead of three times (3X) a day. Three times-a-day milking is currently the most frequently used milking schedule in Iranian Holstein dairy cows, however, four times a day are also rarely applied. The objectives of this study were to estimate variance components for test day (TD) milk yield using a random regression TD model to evaluate the effect of milking frequency (3X vs. 4X) on genetic parameters associated with milk yield in the first and second lactations of Iranian Holstein dairy cows and determines how much milk yield changes with increasing in milking frequency in different lactation yield. It is expected that increasing milking frequency from 3X to 4X causes augmentation of genetic parameters of milk production and also increase in milk production, thereby would be more profitable for farmers.

## Methods

### Data

A total of 142,604 TD records of milk yield were measured on 20,762 first and second lactations of Iranian Holstein dairy cows from 2009 to 2011. The cows were from 113 herds located in the different regions of Iran. In average, for each cow 6 records were available along the lactation period. The records of cows were deleted if they had fewer than 4 or more than 10 TD records or their ages at first calving were below 20 months or above 36 months, further edits excluded irregular data for daily milk yield (<1.0 and >70 kg). Number of times milked per day with 2 levels (3X or 4X). In both 3X and 4X, the cows were milked for 305-d period. Very small number of records were available before day 5 and after day 305 which were discarded. A summary of the pedigree information and the data used are presented in Tables 1 and 2, respectively. The residual variance was considered to be heterogeneous and modeled by grouping days in milk into 10 equal segments of 30 days containing similar variations.

**Table 1:** Summary of the pedigree information of milk yield data in 3X and 4X milking frequency

	Number of					
Trait	Milking frequency	Animal	Records	Sire	Dam	Herd
Milk yield	3	18,764	117,168	1002	16,458	112
	4	2810	25436	296	2198	8

**Table 2:** Descriptive statistics of the test-day records of milk yield (kg) for some selected days in milk with 3X and 4X milking frequency

	3X Milking						4X Milking					
	Lact 1			Lact 2			Lact 1			Lact 2		
DIM	N	Mean	SD	N	Mean	SD	N	Mean	SD	N	Mean	SD
5–35	6392	29.7	4.9	7691	38	6.3	1142	32.5	5	1313	41.8	6.1
36–65	7022	33.4	4.9	8218	40.7	6.4	1298	37.3	4.8	1374	46.1	6.2
66–95	7097	33.9	5.1	8246	40.2	6.6	1292	38.6	4.9	1375	45.8	6.2
96–125	7430	33.4	5	8778	38.6	6.6	1336	38	4.8	1467	43.8	6.2
126–155	6816	32.8	5.3	7815	36.4	6.7	1330	37.2	4.8	1448	41.3	6.1
156–185	6293	32.4	5.4	7057	34.4	6.7	1375	36.4	4.8	1394	38.8	5.9
186–225	5234	31.8	5.6	5456	32.5	6.7	1336	35.4	4.7	1260	36.6	6.3
226–245	4287	31	5.6	4138	30.9	6.8	1324	34.4	4.6	1217	34.2	6.3
246–275	3105	30	5.7	2702	29.1	6.7	1318	39	4.5	1045	31.8	6.2
276–305	1938	29.2	5.8	1453	26.7	6.1	1045	31.9	4.8	747	29.8	6

### Model

A single trait random regression model was applied to estimate genetic parameters of test day (TD) records of milk yield in the first and second lactations. The fixed effects of herd-test date, calving age and the number of days in milk were fitted in the model. Also the additive genetic effects and animal permanent environmental effects were added to the model analysis as the random effects. The data were analysed using ASReml software [15].

The genetic analysis of the milk yields were done using the following model:

$$ {y}_{ijno s}=HT{D}_i+{{\displaystyle \sum_{n=1}^k{b}_n\left( ag{e}_{ijn}\right)}}^n+{\displaystyle \sum_{n=1}^k{c}_n}{\left({ \dim}_{ijno}\right)}^n+{\displaystyle \sum_{n=1}^{k_{\alpha }-1}{\alpha}_{pn}}{\varphi}_n\left({ \dim}_{ijno}\right)+{\displaystyle \sum_{n=1}^{k_{PE}-1}{\gamma}_{pn}}{\varphi}_n\left({ \dim}_{ijno}\right)+{e}_{ijno s} $$

Where: y_*ijnos*_ is the individual test-day records HTD_*i*_ is i^th^ Herd-Test date, b_*n*_ is fixed regression coefficient of age at calving, age_*ijn*_ is calving age, c_*n*_ is fixed regression coefficient of days in milk, dim_*ijno*_ is days in milk, *φ*_*n*_ is n^th^ Legender polynomial for days in milk, *α*_*pn*_ is additive genetic effects, *γ*_*pn*_ is animal permanent environmental effects and e_*ijnos*_ is the residuals.

Models with different order of Legendre polynomials for the additive genetic effects and the animal permanent environmental effects were compared using Schwarz’s Bayesian information criteria (BIC) [29]:

$$ \mathrm{B}\mathrm{I}\mathrm{C}=-2l+K\; log\;n $$

Where *l* is the Log likelihood values, K is the number of estimated parameters and n is the number of observations.

## Results and discussion

The best models for the genetic analyses were selected based on the BIC. Accordingly, in the selected models, the order of fit for genetic and permanent environmental effects, the number of estimated parameters, log likelihood values and BIC are presented in bold Tables 3 and 4.

**Table 3:** Number of estimated parameters, log likelihood values and Schwarz’s Bayesian information criteria (BIC) for 3X milking frequency in the first and second lactations

Lactation	Ka^a^	Kpe^b^	Number of parameters	Log likelyhood	BIC
Lact 1	1	1	16	−106,018.02	212,111.7
	2	1	19	−105,796.05	211,682
	2	2	22	−105,772.28	211,648.6
	3	2	25	−105,735.65	211,589.5
	3	3	28	−105,703.71	211,539.9
	4	2	28	−105,680.26	211,493
	4	3	31	−105,656.98	211,460.6
	4	4	34	−105,645.56	211,451.9
	5	2	31	−105,661.18	211,469
	5	3	34	−105,648.43	211,457.7
	**5**	**4**	**37**	**−105,635.52**	**211,446** ^**c**^
	5	5	40	−105,675.21	211,539.6
Lact 2	1	1	16	−131,871.6	263,819.6
	2	1	19	−131,569.2	263,229.1
	2	2	22	−131,516.12	263,137.3
	**3**	**2**	**25**	**−121,394.82**	**242,909** ^**c**^
	3	3	28	−141,350.47	282,834.7
	4	2	28	−131,347.03	262,827.8
	4	3	31	−131,303.31	262,754.7
	4	4	34	−131,275.85	262,714.1
	5	2	31	−131,134.43	262,416.9
	5	3	34	−131,086.81	262,336
	5	4	37	−131,050.94	262,278.6
	5	5	40	−131,041.94	262,274.9

^a^ka: order of fit for additive genetic effect

^b^ke: order of fit for permanent environmental effect

^c^Selected order of legendre polynomials for the genetic and permanent environmental effects (best model)

**Table 4:** Number of estimated parameters, log likelihood values and Schwarz’s Bayesian information criteria (BIC) for 4X milking frequency in the first and second lactations

Lactation	Ka^a^	Kpe^b^	Number of parameters	Log likelyhood	BIC
Lact 1	1	1	16	−24,329.13	48,723.88
	2	1	19	−24,234.39	48,546.7
	2	2	22	−24,226.59	48,543.4
	3	2	25	−24,174.07	48,450.67
	3	3	28	−24,171.08	48,456.99
	4	2	28	−24,173.33	48,461.49
	4	3	31	−24,169.18	48,465.49
	4	4	34	−24,167.36	48,474.16
	**5**	**2**	**31**	**−24,112.26**	**48,351.65** ^**c**^
	5	3	34	−24,108.09	48,355.62
	5	4	37	−24,100.91	48,353.56
	5	5	40	−24,118.71	48,401.46
Lact 2	1	1	16	−27,021.36	54,108.25
	2	1	19	−26,923.09	53,923.99
	2	2	22	−26,913.57	53,917.24
	3	2	25	−26,859.41	53,821.2
	3	3	28	−26,849.15	53,812.97
	4	2	28	−26,857.37	53,829.41
	4	3	31	−26,849.17	53,825.3
	4	4	34	−26,845.67	53,830.58
	5	2	31	−26,794.93	53,716.82
	**5**	**3**	**34**	**−26,787.09**	**53,713.42** ^**c**^
	5	4	37	−26,784.15	53,719.83
	5	5	40	−26,791.15	53,746.11

^a^ka: order of fit for additive genetic effect

^b^ke: order of fit for permanent environmental effect

^c^Selected order of legendre polynomials for the genetic and permanent environmental effects (best model)

### Phenotypic analysis

**Fig. 1:**
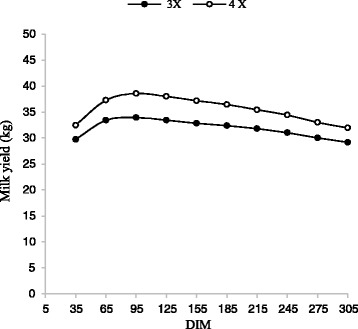
Trend of milk yield by days in milk (DIM) for three and four times milking in first lactation

**Fig. 2:**
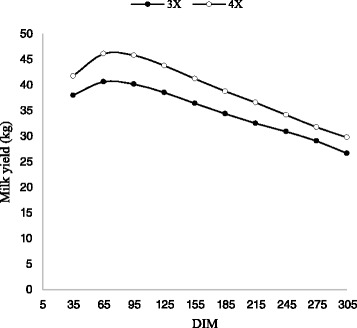
Trend of milk yield by days in milk (DIM) for three and four times milking in second lactation

The average milk yield in the first lactation were 31.8 and 35.5 kg for 3X and 4X milking frequency, respectively. For the second lactations, the values were 34.7 and 39 kg for 3X and 4X milking frequency, respectively. In comparison with 3X milking frequency, 11.6 and 12.2 % greater milk were obtained during the first and second lactations, respectively when 4X was applied (*p* < 0.01). This indicated that the 4X milking frequency is more effective during the second lactation than that of the first lactation. Figures 1 and 2 present the effect of milking frequency on milk yield for the first two lactations in different DIM, which is similar to the normal pattern of the milk production in dairy cows.

For both the first and second lactations, similar patterns were observed in which the greatest milk yield was obtained from day 65 to 155 and thereafter, gradually reduced until the end of lactation period. In addition, the greatest difference (14 %) between milk yield with 3X and 4X milking frequency was obtained at day 95 and 125, for the first and second lactations, respectively. The lowest difference (9 %) between the values for milk yield with 3X and 4X milking frequency was obtained at day 275 and 305, respectively. Similar results were reported by Armstrong [1].

### Estimation of genetic parameters

**Fig. 3:**
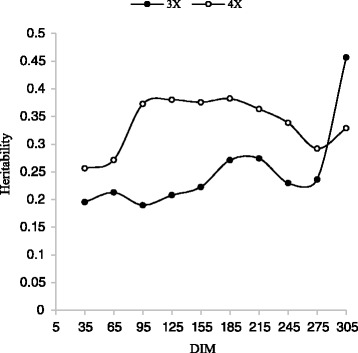
Trajectory of estimated heritability for milk yield by days in milk (DIM) for three and four times milking in first lactation

**Fig. 4:**
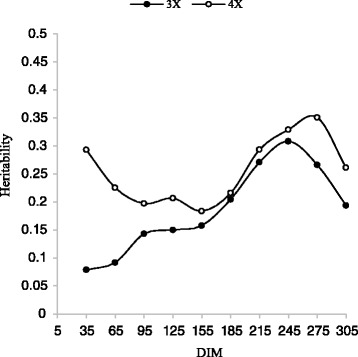
Trajectory of estimated heritability for milk yield by days in milk (DIM) for three and four times milking in second lactation

A range of 0.09 to o.55 has been reported for the heritability of milk yield [5, 10, 27, 34]. Figures 3, 4 and Table 5 show milk yields heritability estimates by DIM for 3X and 4X milking frequency during the first two lactations. The overall heritability estimates for 3X and 4X during the first lactation were 0.25 and 0.19, respectively. The values were 0.34 and 0.26 for the second lactation. The results were in agreement with those reported by Muir et al. [25]. The heritability estimates were slightly higher than the results derived from a multivariate model using the same dataset [28]. The results show the estimated heritabilities of both milking frequency in first lactation were lower than those for the second lactation which is in consistent with the results reported by Miglior et al. [24]. During the lactation periods, the estimated heritability of 4X was higher than 3X (excepted d 305 in first lactation) but the differences between the heritability of 3X and 4X in the first lactation were higher than those in the second lactation (Figs. 3 and 4). The changes in heritabilities are because of the changes in the variance components which are explained in the following paragraphs.

**Table 5:** Additive genetic (*σ* ^*2*^_*a*_), animal permanent environmental (*σ* ^*2*^_*pe*_), residual (*σ* ^*2*^_*e*_) variances and heritability (h^2^) of the test-day records of milk yield (Kg) for some selected days in milk and different milking frequency

Variances	Days in milk (DIM)									
	5–35	36–65	66–95	96–125	126–155	156–185	186–215	216–245	246–275	276–305
Lact 1 (3X)										
*σ* ^*2*^_*a*_	5.0	5.7	4.9	5.2	6.1	8.1	9.1	8.0	8.5	21.7
*σ* ^*2*^_*pe*_	9.2	11.0	11.7	11.3	11.9	13.4	15.2	17.8	20.0	18.1
*σ* ^*2*^_*e*_	11.4	10.0	9.1	8.5	9.4	8.5	8.9	9.2	7.5	7.7
*h* ^*2*^	0.20	0.21	0.19	0.21	0.22	0.27	0.27	0.23	0.24	0.46
Lact 2 (3X)										
*σ* ^*2*^_*a*_	4.0	4.1	6.3	6.8	7.3	9.9	14.0	16.4	14.7	12.3
*σ* ^*2*^_*pe*_	25.5	21.7	22.3	24.2	25.6	25.7	25.1	25.4	29.3	40.9
*σ* ^*2*^_*e*_	20.9	19.0	15.7	14.3	13.4	12.7	12.5	11.5	11.2	10.1
*h* ^*2*^	0.08	0.09	0.14	0.15	0.16	0.20	0.27	0.31	0.27	0.19
Lact 1 (4X)										
*σ* ^*2*^_*a*_	10.1	8.9	12.0	11.6	11.5	11.2	11.0	9.5	9.1	13.5
*σ* ^*2*^_*pe*_	20.3	14.3	11.6	10.7	10.6	10.9	11.4	12.7	15.6	21.6
*σ* ^*2*^_*e*_	8.9	9.5	8.6	8.3	8.5	7.3	7.8	6.0	6.5	6.0
*h* ^*2*^	0.26	0.27	0.37	0.38	0.38	0.38	0.36	0.34	0.29	0.33
Lact 2 (4X)										
*σ* ^*2*^_*a*_	16.6	12.0	10.4	10.0	8.7	10.2	14.4	16.6	19.2	15.5
*σ* ^*2*^_*pe*_	18.5	25.0	27.2	26.9	26.1	25.8	25.8	25.9	27.6	37.2
*σ* ^*2*^_*e*_	21.6	16.4	15.2	11.6	12.3	11.4	8.9	8.0	7.9	6.7
*h* ^*2*^	0.29	0.23	0.20	0.21	0.18	0.22	0.29	0.33	0.35	0.26

Variances tended to be larger at the beginning and the end of lactation, which is probably because of the smaller number of records that corresponded to these time periods, and are possible artifacts of Legendre polynomials.

**Fig. 5:**
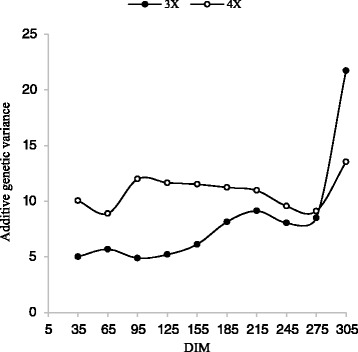
Trajectory of additive genetic variance for milk yield by days in milk (DIM) for three and four times milking in first lactation

**Fig. 6:**
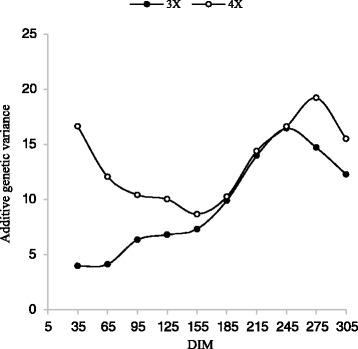
Trajectory of additive genetic variance for milk yield by days in milk (DIM) for three and four times milking in second lactation

For the first lactation, additive genetic variances (V_A_) of 3X increased slowly and for and 4X decrease slowly during the lactation trajectory (Fig. 5). Additive genetic variance of 3X in the second lactation was decrease sharply from day 5 to 185 and then increased slowly up to 275 days. Additive genetic variance of 3X in the second lactation was increase sharply from day 5 to 245 and then decrease to end of lactation (Fig. 6).

**Fig. 7:**
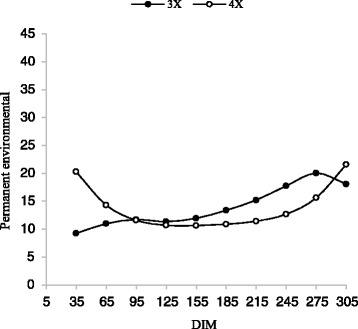
Trajectory of animal permanent environmental variance for milk yield by days in milk (DIM) for three and four times milking in first lactation

**Fig. 8:**
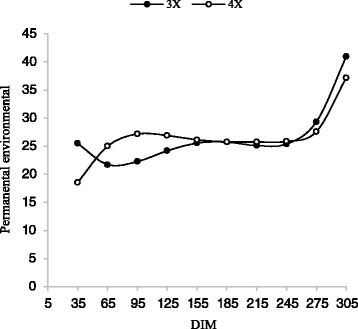
Trajectory of animal permanent environmental variance for milk yield by days in milk (DIM) for three and four times milking in second lactation

As indicated in Table 5, for all traits, animal permanent environmental variances increased slowly during the lactation period (Figs. 7 and 8). Based on the results of Meyer et al. [23] and Zavandilova et al. [35] on Holstein cows, the highest additive genetic and permanent environment variances for dairy traits occurred in the first and last days of lactation.

**Fig. 9:**
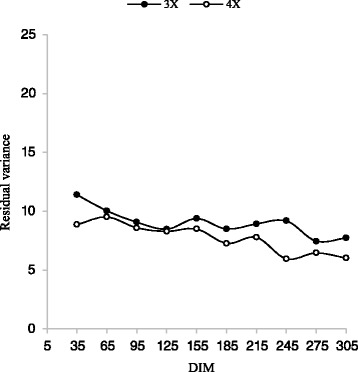
Trajectory of residual variance for milk yield by days in milk (DIM) for three and four times milking in first lactation

**Fig. 10:**
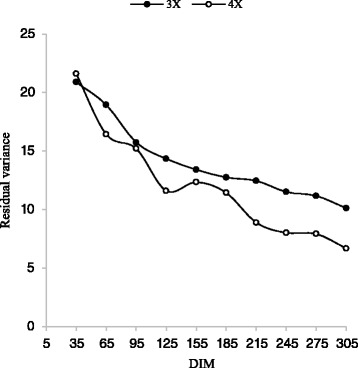
Trajectory of residual variance for milk yield by days in milk (DIM) for three and four times milking in second lactation

In general, the trends in the V_A_ and V_EP_ throughout the lactations obtained in this study are comparable to trends found by Druet et al. [12] and Strabel et al. [35]. For all traits, except for 3X milking frequency in the first lactation, residual variance was smaller, compared with the total phenotypic variance. This indicates a good fit of the model. Since residual variance related to additive genetic variances was larger for 3X milking frequency in the first lactation, therefore, the model should be modified, so that the genetic parameters could be estimated with a greater accuracy (Figs. 9 and 10). Residual variances decreased slowly during the lactation for all traits; therefore, heritabilities increased slowly during the lactation. These results are similar to those observed by Cobuci et al. [8] and Biassus et al. [2].

Table 6 shows the genetic and phenotypic correlations for milk yield along DIM for the different milking frequency in first two lactations. The genetic correlations for 3X cow ranged from −0.18 to 0.97 for the first lactation, from 0.17 to 0.99 for the second lactation and for 4X cow from −0.71 to 0.97 and from −0.22 to 0.97 for first and second lactation respectively. In general, genetic correlations declined with an increasing interval in DIM for both lactations. Similar results have been reported by El Faro et al. [13] for Caracu cattle. Karacao et al. [20] reported genetic correlations for milk yield at calving to midlactation monotonically decreased to 0.40 and at the end of the lactation, decreased to −0.06 also genetic correlation between d 1 and 247 was estimated to be 0.03.

**Table 6:** Estimates of genetic (below diagonal) and phenotypic (above diagonal) correlations between milk yield on selected days in milk (DIM) for different milking frequency in the first and second lactations

MF^a^ Lactation and DIM	DIM										
		35	65	95	125	155	185	215	245	275	305
3X First											
35		-	0.34	0.29	0.27	0.23	0.19	0.17	0.18	0.19	0.10
65		0.19	-	0.59	0.51	0.46	0.47	0.47	0.43	0.38	0.40
95		0.14	0.87	-	0.62	0.57	0.55	0.52	0.50	0.46	0.41
125		0.17	0.67	0.94	-	0.64	0.60	0.56	0.53	0.50	0.42
155		0.07	0.74	0.95	0.97	-	0.66	0.62	0.56	0.51	0.49
185		−0.09	0.86	0.94	0.87	0.95	-	0.70	0.63	0.55	0.59
225		−0.18	0.89	0.94	0.81	0.88	0.97	-	0.69	0.63	0.63
245		−0.14	0.76	0.92	0.82	0.80	0.83	0.91	-	0.73	0.62
275		0.03	0.54	0.80	0.81	0.72	0.65	0.71	0.92	-	0.63
305		0.02	0.76	0.72	0.64	0.75	0.84	0.80	0.63	0.53	-
3X Second											
35		-	0.51	0.43	0.38	0.35	0.33	0.31	0.29	0.27	0.22
65		0.52	-	0.58	0.55	0.51	0.44	0.38	0.32	0.27	0.23
95		0.29	0.96	-	0.65	0.61	0.55	0.46	0.39	0.32	0.26
125		0.32	0.90	0.97	-	0.68	0.63	0.56	0.48	0.40	0.30
155		0.45	0.73	0.79	0.92	-	0.70	0.66	0.59	0.50	0.36
185		0.53	0.47	0.50	0.70	0.92	-	0.73	0.69	0.60	0.44
225		0.54	0.27	0.28	0.50	0.80	0.97	-	0.75	0.69	0.52
245		0.51	0.18	0.18	0.41	0.73	0.93	0.99	-	0.76	0.63
275		0.43	0.20	0.23	0.44	0.73	0.91	0.96	0.98	-	0.75
305		0.17	0.37	0.46	0.60	0.72	0.74	0.71	0.73	0.85	-
4X First											
35		-	0.26	0.19	0.24	0.25	0.20	0.14	0.14	0.17	0.04
65		−0.71	-	0.67	0.55	0.49	0.51	0.50	0.45	0.33	0.37
95		−0.57	0.90	-	0.68	0.62	0.61	0.58	0.54	0.45	0.43
125		−0.18	0.67	0.90	-	0.71	0.68	0.61	0.58	0.52	0.45
155		0.02	0.61	0.82	0.97	-	0.71	0.65	0.61	0.54	0.48
185		−0.01	0.75	0.83	0.90	0.95	-	0.73	0.68	0.58	0.55
225		−0.11	0.85	0.84	0.81	0.86	0.96	-	0.73	0.63	0.59
245		−0.09	0.75	0.80	0.79	0.80	0.86	0.94	-	0.74	0.63
275		0.00	0.47	0.68	0.79	0.76	0.72	0.75	0.91	-	0.67
305		−0.41	0.67	0.74	0.72	0.73	0.77	0.73	0.61	0.55	-
4X Second											
35		-	0.49	0.47	0.45	0.35	0.24	0.20	0.26	0.32	0.17
65		0.40	-	0.67	0.60	0.54	0.49	0.45	0.39	0.31	0.30
95		0.36	0.87	-	0.71	0.65	0.57	0.50	0.44	0.38	0.32
125		0.31	0.61	0.91	-	0.73	0.64	0.56	0.52	0.47	0.37
155		0.14	0.57	0.86	0.95	-	0.71	0.66	0.61	0.54	0.45
185		−0.04	0.63	0.72	0.70	0.87	-	0.76	0.71	0.60	0.55
225		0.01	0.63	0.61	0.53	0.72	0.96	-	0.80	0.69	0.63
245		0.27	0.55	0.56	0.55	0.70	0.86	0.93	-	0.80	0.69
275		0.41	0.32	0.46	0.59	0.69	0.71	0.75	0.93	-	0.74
305		−0.29	0.17	0.29	0.42	0.73	0.97	0.97	0.88	0.73	-

^a^Milking frequency

The phenotypic correlations were smaller than the corresponding genetic correlations, but they followed a pattern similar to the corresponding genetic correlations for all traits (Table 6). The least phenotypic correlations between 5 and 305 DIM, were 0.10 and 0.22 for 3X cow and 0.04 and 0.17 for 4X cow for the first and second lactations, respectively.

Negative phenotypic and genetic correlations were observed between initial and final test-days. After calving, the cow suffers from post-calving stress and also from an energy deficit. This can be caused the negative values. Negative genetic correlations estimated by RRM using different functions have also been reported for Holstein cattle by Jamrozik and Schaeffer [19], Olori et al. [26], Brotherstone et al. [6] and Kettunen et al. [21], and in Brazil by Cobuci et al. [7], Costa et al. [9] and Bignardi et al. [3, 4]. Jakobsen et al. [18] reported genetic correlations estimates higher than 0.40 for first lactation test-day milk yield of Holstein dairy cattle, therefore slowly lower than some estimates observed in this study. However, lower estimates, even close to zero, were obtained for genetic correlations between test-day milk yields in first lactation by Cobuci et al. [7] and Biassus et al. [2].

## Conclusion

The results of this study show the milk yield in the first and the second lactations is significantly affected by milking frequency. The results also indicate that the milk yield is more affected by milking frequency in the middle of the lactation periods. In the first lactation, the Phenotypic and genetic correlations between the 3X milk yields in the studied DIM were higher than those for the 4X while they were similar in the second lactation. In both lactations, the heritability estimated for milk yield in 3X was smaller than those for 4X, therefore the 4X cows could be genetically more accurately evaluated. However, because of the cost of other inputs like labor and failure in the marketing, the application of 4 times milking per day in Iranian Holstein cows should be investigated economically.
